# Developing a system that can automatically detect health changes using transfer times of older adults

**DOI:** 10.1186/s12874-016-0124-4

**Published:** 2016-02-20

**Authors:** Greet Baldewijns, Stijn Luca, Bart Vanrumste, Tom Croonenborghs

**Affiliations:** KU Leuven Technology Campus Geel, AdvISe, Kleinhoefstraat 4, Geel, Belgium; KU Leuven, ESAT-STADIUS,, Leuven, Belgium; iMinds Medical Information Technology Department, Leuven, Belgium; KU Leuven, Department of Computer Science, DTAI, Leuven, Belgium; Program in Translational NeuroPsychiatric Genomics, Brigham and Women’s Hospital, Harvard Medical School, Broad Institute of Massachusetts Institute of Technology and Harvard, Cambridge, MA, USA

**Keywords:** Assisted living, Gerontechnology, Change detection algorithms, Statistical process control, Log logistic distributions, Gait speed

## Abstract

**Background:**

As gait speed and transfer times are considered to be an important measure of functional ability in older adults, several systems are currently being researched to measure this parameter in the home environment of older adults. The data resulting from these systems, however, still needs to be reviewed by healthcare workers which is a time-consuming process.

**Methods:**

This paper presents a system that employs statistical process control techniques (SPC) to automatically detect both positive and negative trends in transfer times. Several SPC techniques, Tabular cumulative sum (CUSUM) chart, Standardized CUSUM and Exponentially Weighted Moving Average (EWMA) chart were evaluated. The best performing method was further optimized for the desired application. After this, it was validated on both simulated data and real-life data.

**Results:**

The best performing method was the Exponentially Weighted Moving Average control chart with the use of rational subgroups and a reinitialization after three alarm days. The results from the simulated data showed that positive and negative trends are detected within 14 days after the start of the trend when a trend is 28 days long. When the transition period is shorter, the number of days before an alert is triggered also diminishes. If for instance an abrupt change is present in the transfer time an alert is triggered within two days after this change. On average, only one false alarm is triggered every five weeks. The results from the real-life dataset confirm those of the simulated dataset.

**Conclusions:**

The system presented in this paper is able to detect both positive and negative trends in the transfer times of older adults, therefore automatically triggering an alarm when changes in transfer times occur. These changes can be gradual as well as abrupt.

## Background

Although ageing is often associated with a decline in health, most older adults want to live in their own home environment as long as possible. Automated homecare systems that can help older adults maintain their independence are therefore in high demand [1–3] and the development of these systems is receiving a lot of research attention. Research groups aim to develop systems which can automatically monitor the health of older adults enabling the detection of both acute events, such as fall incidents, and gradual changes in health or functional ability [4].

Because a decline in gait speed has a predictive value for a broad array of adverse events such as physical functional decline [5–8], cognitive impairment [9–12] and fall incidents [7, 9, 10, 13–15] it is one of the parameters often monitored in these systems. For the monitoring of gait speed as well as the closely related transfer times (the time needed to cross a predefined transfer zone) both wearable sensors such as accelerometers and gyroscopes [11, 16, 17] and contactless sensors, such as motion detection systems [18], radar [19] and cameras [20, 21] are used. Although these systems provide accurate measurements for healthcare workers, the majority of this research does not further process this data. Consequently, the data still needs to be reviewed for each patient individually, which can be very time-consuming and not feasible for healthcare workers who have a high number of patients. Additionally, specialized knowledge is needed to interpret these measurements.

The goal of our research therefore was the development of a system that could automatically detect deviating trends in transfer times of older adults. When a deviating trend is detected, an alarm is triggered alerting the healthcare worker. Said triggering of an alarm will reduce the time healthcare workers need to spend on reviewing the data. These alarms will furthermore allow for quicker interventions, before major health problems arise, and will consequently improve the quality of care and quality of life of older adults.

To automatically detect these deviations in the transfer times of older adult this study suggests the use of Statistical Process Control (SPC) techniques. Three statistical process control techniques: Tabular CUmulative SUM (CUSUM) chart, Standardized CUSUM and Exponentially Weighted Moving Average (EWMA) chart, were evaluated on simulated data. The best performing SPC method was selected and optimized to suit the application. After optimization the results were extensively validated on both simulated as well as on real-life datasets showing that chosen SPC technique is well-suited to detect gradual changes in the transfer times of older adults.

Although in previous studies SPC techniques are sometimes used to predict future events when monitoring health-related variables [22, 23], the use of SPC techniques for the monitoring of transfer times is, however, new. We published a first proof of concept in which the basic SPC techniques were tested on a limited set of simulated data in [24].

The remainder of the paper is structured as follows. First, a general description of the evaluated statistical process control methods is provided. Thereafter, the different simulated datasets for both training and validation, as well as the real-life dataset are discussed. This is followed by the empirical evaluation of the different SPC techniques. Finally, an in-depth discussion of the results and a general conclusion is presented. An overview of the different abbreviations used in the paper and their meaning is given at the end of the paper.

## Methods

In this section first the different datasets used to evaluate and optimize the different statistical process control techniques are discussed. This is followed by a general description of statistical process control and the used control charts. Lastly, the experimental set-up is discussed.

### The dataset

Four real-life datasets were acquired during previous research [20, 21]. This limited number of datasets was, however, deemed insufficient to evaluate the different SPC techniques as well as to optimize and validate the best performing method. Simulated data were thus generated for evaluation, optimization and a first validation. The real-life dataset was used for further validation of the optimized technique.

#### Real-life data

**Fig. 1:**
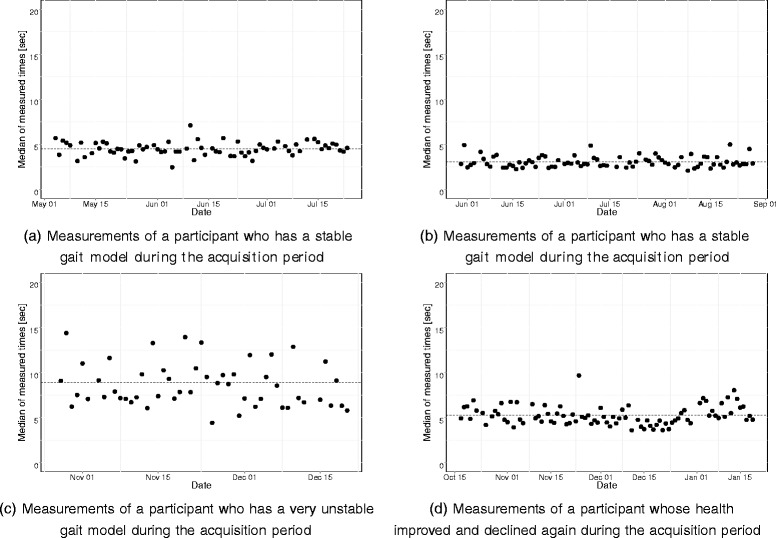
Medians of measured transfer times per day of the different participants. The dotted line is the average of the measured transfer times

To acquire real-life data, four camera systems consisting of multiple wall-mounted webcams, were installed in the homes of four older adults for two to three months. From the resulting video data transfer times (the time a person needs to cross a predefined transfer zone) were measured. To reduce the influence of noise on the system a median of the measured transfer times was calculated per day, thus resulting in four datasets of medians of measured transfer times. An in-depth discussing of the measured transfer times is given in [21]. During this previous study it was shown that a person in good health also had a stable gait model which is defined by short transfer times and small fluctuations in the measured times (see Fig. 1(a) and (b)). It was also shown that a person with different health issues (e.g. high fall risk, cognitive problems, etc.) had an unstable gait model which is identified by long transfer times and large fluctuations in the measured times (see Fig. 1(c)). Lastly, it was also observed that an improving health presents itself in the shortening of the transfer time and a smaller variability in these transfer times. A declining health in turn was visible by an increase in the transfer time and a larger variability (see Fig. 1(d)). It is therefore important that the designed method is able to detect deviating trends without triggering alerts when ’normal’ fluctuations in the transfer times occur.

Ethical approval for the study was provided by the Medical Ethics Committee of the Leuven University Hospitals (Trial registration numbers ML9820 and S55834 Registered 18 October 2013) and a written informed consent was obtained from all participants for both the data acquisition as well as for the publication of individual results.

More in-depth information concerning the algorithm used to automatically detect and time the transfer zones and the acquired data can be found in [21].

#### Simulated data generation

There are several aspects that were taken into account when generating the simulated transfer times. First of all, the type of distribution used to generate the transfer times was determined. This was done based on the real-life transfer times. Several distributions types: Normal, Log-Normal, Gamma, Nakagami, Logistic, Log-Logistic, Birnbaum-Saunders and Weibull, were selected based on visual inspection of the different datasets. To assess which of these distributions was the best fit, the parameters for each distribution and for each dataset were estimated using maximum likelihood estimation [25]. Subsequently, the Kolmogorov-Smirnov test was used to determine the probability that the evaluated distribution matched with the data [26]. The distribution with the highest average *P*-value (see Table 1) was the log-logistic distribution. This distribution is therefore used to generate the simulated data.

**Table 1:** Average and standard deviation of the *P*-values resulting from the Kolmogorov-Smirnov test for the four real-life datasets

Distribution	Mean	Standard deviation
Gamma	0.2445	0.1295
Log-Normal	0.5062	0.2342
Normal	0.2649	0.2417
Nakagami	0.3368	0.2362
Birnhaum-Saunders	0.4050	0.2213
Logistic	0.3169	0.1605
**Log Logistic**	**0.8140**	**0.1454**
Weibull	0.1362	0.1055

The distribution with the highest average P-value is marked in bold

The Log-Logistic distribution is a continuous probability distribution for a non-negative random variable. It is the probability distribution of a random variable whose logarithm has a logistic distribution [27]. The probability density function (pdf) is defined through

(1) $$  p(x|\mu,\sigma)=\frac{1}{\sigma}\frac{1}{x}\frac{e^{z}}{\left(1+e^{z}\right)^{2}}; x\geq 0  $$

and

(2) $$  z=\frac{log(x) - \mu}{\sigma}  $$

with

- *μ* = the location parameter
- *σ* = the scale parameter
- *x* = transfer time.

**Fig. 2:**
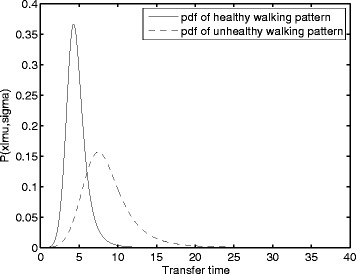
Probability density function for both stable and unstable gait models

Using maximum likelihood estimation, *μ* and *σ* were estimated for a stable gait model based on the data in Fig. 1(b). As an unstable gait model typically has longer transfer times and more variability [21], *μ* and *σ* of an unstable gait model were therefore determined using this knowledge. A stable model was therefore defined by a small value for both *μ* and *σ* an unstable gait model in turn was defined by an increase in both *μ* and *σ*. The resulting probability density functions are shown in Fig. 2. Additionally, two models were defined combining the properties of both the stable gait model and the unstable gait model. An overview of the different parameters for each model is given in Table 2.

**Table 2:** Parameter values of the simulated data models

	*σ*=0.155	*σ*=0.206
*μ*=1.504	SGM^a^	TGM1^b^
*μ*=2.097	TGM2^c^	UGM^d^

Notes

A gait model is defined as a combination of

an average transfer time and standard deviation

^a^Stable Gait Model

^b^Theoretical Gait Model 1 (not based on real-life data)

^c^Theoretical Gait Model 2 (not based on real-life data)

^d^Unstable Gait Model

To determine the number of measurements on a certain day, a poisson distribution was used. Poisson distributions are typically used to express the probability of a given number of events occurring in a fixed interval of time [28], which is a good match with our application. The probability density function used to sample the number of measurements per day is:

(3) $$ P_{N}(n|\lambda)=\frac{\lambda^{n}}{n!}e^{-\lambda}  $$

with *λ*=5 chosen as the mean of the distribution.

Lastly, the method used to alter from a stable gait model to an unstable gait model and vice versa was implemented. During the transition period, the parameters of the gait model were determined using linear interpolation between the original and the new model. For each day in the transition period, a new set of distribution parameters was calculated.

The simulated data generator was built using MathWorks’ Matlab. The code will become available for download.

#### Simulated trainings dataset

To select and optimize the best method, three different types of simulation scenarios were chosen based on those observed in the real-life dataset: no trend present, one trend present and two trends present. For each type, two different scenarios were defined. An overview can be found in Table 3, one column corresponds with one scenario. Transfer time data for each possible simulation scenario was generated 20 times, resulting in 120 different datasets in total.

**Table 3:** Overview of the different training simulation scenarios

Transition Scenario^a^	No trend		One trend		Two trends	
	*T* *r*_*S*_	*T* *r*_*U*_	*T* *r*_*SU*_	*T* *r*_*US*_	*T* *r*_*SUS*_	*T* *r*_*USU*_
Duration	12 weeks		28 weeks		44 weeks	
Model 1^b^	SGM	UGM	SGM	UGM	SGM	UGM
Model 2			UGM	SGM	UGM	SGM
Model 3					SGM	UGM
Transition 1 length			4 weeks		4 weeks	
Transition 2 length					4 weeks	

Notes

One column corresponds with one scenario

^a^ *Tr* stands for Training scenario, _*S*_ stands for Stable gait and _*U*_ stands for Unstable gait

^b^SGM stands for Stable Gait Model and

UGM stands for Unstable Gait Model

#### Simulated validation dataset

To validate the resulting method, an extended validation dataset was generated. The goal of this dataset was firstly, to validate the results acquired with the training set and secondly, to find the limitations of the selected method. To validate the results on the training set, the basic scenarios similar to the training set were generated. Additionally, the basic scenarios were generated with different transition lengths (0,4,8 and 12 weeks). Moreover, transition scenarios were also generated with a change in either *μ* or *σ*, resulting in transitions to TGM1 and TGM2 as defined in Table 2. Lastly, the number of measurements per day was halved. Each additional scenario type was simulated 20 times and was 52 weeks long resulting in 520 different validation simulation scenarios. An overview of the different simulation scenarios is given in Table 4, again one column corresponds with one scenario.

**Table 4:** Overview of the different validation simulation scenarios

Transition^a^ Scenario	No trend		One trend						Two trends					
	*V*_*S*_	*V*_*U*_	*V*_*SU*_	*V*_*ST*1_	*V*_*ST*2_	*V*_*US*_	*V*_*UT*1_	*V*_*UT*2_	*V*_*SUS*_	*V*_*ST*1*S*_	*V*_*ST*2*S*_	*V*_*USU*_	*V*_*UT*1*U*_	*V*_*UT*2*U*_
Duration	52 weeks		52 weeks						52 weeks					
Model 1^b^	SGM	UGM	SGM	SGM	SGM	UGM	UGM	UGM	SGM	SGM	SGM	UGM	UGM	UGM
Model 2			UGM	TGM1	TGM2	SGM	TGM1	TGM2	UGM	TGM1	TGM2	SGM	TGM1	TGM2
Model 3									SGM	SGM	SGM	UGM	UGM	UGM
Transition 1			0/4/8/12	4	4	0/4/8/12	4	4	0/4/8/12	4	4	0/4/8/12	4	4
length			weeks	weeks	weeks	weeks	weeks	weeks	weeks	weeks	weeks	weeks	weeks	weeks
Transition 2									0/4/8/12	4	4	0/4/8/12	4	4
length									weeks	weeks	weeks	weeks	weeks	weeks

Notes

One column corresponds with one scenario

^a^ *V* stands for Validation scenario, _*S*_ stands for Stable gait and _*U*_ stands for Unstable gait

^b^SGM stands for Stable Gait Model and UGM stands for Unstable Gait Model

### SPC techniques

SPC techniques are commonly used to check a process during its run and trigger an alert when variations, not inherent to the process, occur using control charts. In our research, control charts are consequently used to detect when one gait model transitions to another one which is consistent with the detection of small deviations from the first gait model. As the CUSUM and EWMA control charts are best fitted [29] the desired application, the remainder of this paper therefore focuses solely on CUSUM and EWMA control charts.

Typically a control chart has three lines: the Upper Control Limit (UCL), Lower Control Limit (LCL) and the Centre Line (CL). From the medians of the transfer times a sample statistic is calculated and subsequently plotted on the control chart. When a deviation occurs, the sample statistic is plotted outside the control limits and an alarm is triggered. To determine the range of natural variation in the transfer times, an initialization period of 14 days was defined. The mean of the measurements conducted in this period is used as the Central Line and a multiple of the standard deviation is used to define the Upper and Lower Control Limits.

#### CUSUM

CUSUM charts calculate the cumulative sum of the deviations of the observations from the target value. The deviations above the target value are accumulated in the positive CUSUM, whereas the deviations below the target value are accumulated in the negative CUSUM. Using this method, the information contained in the current time point and in the previous time points is taken into account, thus facilitating the detection of smaller shifts [29]. In our application one time point coincides with the median of the transfer times measured during one day.

When the transfer times remain stable at the target value, both the positive and negative CUSUM remain around zero. However, when the transfer time increases, a positive drift will develop in the positive CUSUM. If a trend develops in the medians of the transfer times, one of the Control Limits will be exceeded and an alarm will be triggered.

The literature differentiates between Tabular CUSUM and Standardized CUSUM. Both are therefore incorporated in this study.

#### Tabular CUSUM

With the Tabular CUSUM, the positive and negative CUSUM values are calculated using formulas:

(4) $$  C_{i}^{+}=max\left[0,x_{i}-(\mu_{0}+K)+C_{(i-1)}^{+} \right]  $$

and

(5) $$  C_{i}^{-}=max\left[0,(\mu_{0}-K)-x_{i}+C_{(i-1)}^{-} \right].  $$

In both formulas, *μ*_0_ is defined as the target value, which in our application corresponds to the mean of the measured transfer times during the initialisation period and *x*_*i*_ is the current time point. K refers to the allowance or the slack value of *μ*_0_ and is often chosen halfway between the target value and the out of control value [29]. In our application K is expressed as a multiple of the standard deviation of the transfer times measured during the initialisation period:

(6) $$  K=\frac{k}{2}\sigma.  $$

As seen in formulas (4) and (5), both positive and negative CUSUMs accumulate deviations from the target value that are greater than K. If either exceeds the control limits:

(7) $$  UCL = LCL =h\sigma  $$

an alarm is triggered. Both h and k are parameters that need to be set for an effective detection.

#### Standardized CUSUM

Standardized CUSUM uses similar formulas to those of the Tabular CUSUM chart. The value of *x*_*i*_, however, is first standardized using

(8) $$ y_{i}=\frac{x_{i} - \mu_{0}}{\sigma}.  $$

After this standardization, the CUSUM chart is applied on these standardized values.

#### EWMA

The Exponentially Weighted Moving Average (EWMA) control chart is an alternative to the CUSUM chart when one is interested in detecting small shifts [29]. It accumulates the exponentially weighted moving average of all prior sample means. The samples are weighted in decreasing order so that the most recent sample gets the highest weight while the most distant samples contribute very little. The exponentially weighted moving average is calculated as

(9) $$  z_{i}=\lambda x_{i}+(1-\lambda) z_{(i-1)}  $$

with *λ* the weighing factor chosen between 0 and 1. The starting value of *z*_0_ is chosen the same as the central value *μ*_0_.

Upper control limit and lower control limit are calculated as

(10) $$  UCL = \mu_{0}+L\sigma\sqrt{\frac{\lambda}{2-\lambda}\left[1-(1-\lambda)^{2i}\right]}  $$

and

(11) $$  LCL = \mu_{0}-L\sigma\sqrt{\frac{\lambda}{2-\lambda}\left[1-(1-\lambda)^{2i}\right]}.  $$

In these formulas, L determines the width of the control limits. Note that the term 1−(1−*λ*)^2*i*^ will become one when *i* gets larger. The Control Limits will therefore reach a steady state after several days. However, for small values of *i* this term will reduce the width of the Control Limits facilitating the detection of a process deviation immediately after the EWMA chart is started. Similar to both CUSUM control charts, *μ*_0_ corresponds with the mean of the measured transfer times during the initialization period and *σ* with the standard deviation of the same period.

#### Rational Subgroups

In literature a rational subgroup typically is a group of samples which are collected under the same set of conditions. The variation between the samples in the same subgroup is similar to the inherent variation of the process [29]. As the median per day is used as input for the control charts a form of rational subgroups is already implemented in the basic methods. If however there is a difference in the number of measurements within a subgroup, which is the case in our application, Montgomery et al. advise to take this number into account. Within the suggested methodology the number of measurements is used to determine the width of the control limits [29]. This is done in the CUSUM-based methods by dividing K, UCL and LCL with ${\sqrt {n_{i}}}$ and in the EWMA-based methods by dividing L with ${\sqrt {n_{i}}}$, where *n*_*i*_ is the number of measurements that are used to calculate the median per day.

#### Reinitialization

Multiple trends can present themselves subsequently in the transfer times. As the control charts are initialized based on the first two weeks of acquisition data, a subsequent trend could remain undetected. To ensure the detection of any subsequent trends, the control charts can be reinitialized after three consecutive alarm days. This reinitialization is done by replacing the mean and standard deviation of the measured transfer times calculated during initialization period, with those calculated during the 14 days prior to the last alarm day. When the first trend is detected, the control chart will therefore be reinitialized based on the more recent data, facilitating the detection of any subsequent trends.

### Experimental set-up

The previously described SPC techniques were evaluated on the training set. From this evaluation, the two best performing methods were optimized for the desired application. Next, the validation data was used to validate the training set results and the best performing method was selected. This was followed by an assessment of the limits of the chosen method.

#### General approach

To assess if the different methods can successfully detect transitions in the data during the transition period, three evaluation criteria were selected. These criteria were calculated over multiple datasets of the same type (e.g. all the simulation datasets containing a SGM to UGM), and are as follows:

1. Detection Rate (DR)
  The Detection Rate is the percentage of the detected transitions. For our application, it is necessary that the number of transitions that are not detected is kept as low as possible. Ideally, all transitions trigger an alert (=100 % Detection Rate).
2. Average Run Length (ARL)
  Montgomery et al. defined the ARL of a control chart as the number measurements or subgroups needed to detect a transition [29]. In our application this corresponds with the number medians and thus the number of days which pass between the start of a transition and the detection of this transition. This time needs to be kept as short as possible to enable healthcare workers to respond quickly to changes in health. If there is no detection, no ARL is calculated.
3. Average number of false alerts per week (FPR)
  This is the average number of alerts triggered during one week for one person when there is no transition. As the median per day is used as input of the control charts only one false alert can be triggered per day. The number of measurements per day or per week do not influence this number.

If the sample statistic is plotted outside the control limits for more than two consecutive days this is counted as one alarm. An alarm is considered correct when it occurs during the transition period. An alarm is a false alarm when it presents itself at least two days prior to the transition period or after the transition period. One exception was applied to these rules in simulation scenarios where a sudden change in gait model was present (a transition period of 0 days). In this case an alarm was deemed correct when it occurred within 4 days after the sudden change. Before or after this 4 day period, an alarm is deemed as a false alarm.

#### Evaluation of the different SPC techniques

The training set was used to evaluate the performance of the different SPC techniques. The parameters for both CUSUM and EWMA charts were chosen based on a rule of thumb when aiming for a fast detection of small changes with $k=\frac {1}{2}$ and *h*=3 for both types of CUSUM charts and with *λ*=0.15 and *L*=3 for the EWMA chart [29].

#### Optimization

After the evaluation of the different SPC techniques, the two top-ranked methods were selected. Subsequently, the parameters of these methods were further optimized. This was done by calculating DR, ARL and FPR for the training set using a wide variety of parameter combinations. For the CUSUM-based method *k* varied between 0 and 1, and *h* varied between 2 and 4. For the EWMA based method *λ* varied between 0 and 1, and *L* varied between 2 and 4.

After this, the mean of the DR, ARL and FPR was calculated for each possible combination of both parameters over the different simulation scenarios. This was subsequently plotted in a ROC-like plot with DR and ARL on the X- and Y-axis, adding color information for the number of false alarms. The operating point with the best possible combination for all three criteria, giving higher importance to DR and ARL, was subsequently determined based on this plot. After determining the new operating point and comparing the results for both top-ranked methods, the best performing method was selected.

#### Validation & experimental methodology

The validation of the best method was performed in three stages. First, the results after optimization were compared to those on a validation set consisting of similar scenarios as the training set. After this the following items were investigated:

- The effect of a variation in the transition length on the detection results;
- The effect of a change in simulation parameters on the detection results;
- The effect of a reduction in the average number of measurements per day on the detection results;
- The effect of the initialization period length on the detection results.

Lastly, the optimized method was validated on the real-life dataset.

## Results

### Evaluation of the different SPC techniques

The results of evaluating the different SPC techniques are given in Table 5. From this table the following observations can be made:

**Table 5:** Results Tabular CUSUM, Standardized CUSUM and EWMA (when the dataset contains two trends both trends are evaluated separately)

	Tabular CUSUM (TC)								Standardized CUSUM (SC)								EWMA (E)							
	TC		RS^b^		RA^c^		RSRA^d^		SC		RS^b^		RA^c^		RSRA^d^		E		RS^b^		RA^c^		RSRA^d^	
Average number of false positive alerts per week																								
	mean	sd	mean	sd	mean	sd	mean	sd	mean	sd	mean	sd	mean	sd	mean	sd	mean	sd	mean	sd	mean	sd	mean	sd
*T* *r*_*S*_^a^	0.01	0.04	0.06	0.08	0.10	0.04	0.04	0.04	0.19	0.16	0.10	0.03	0.09	0.02	0.19	0.14	0.00	0.00	0.14	0.08	0.00	0.00	0.03	0.06
*T* *r*_*U*_	0.06	0.08	0.09	0.06	0.11	0.08	0.15	0.12	0.13	0.11	0.15	0.11	0.07	0.05	0.18	0.17	0.00	0.00	0.20	0.15	0.00	0.00	0.05	0.06
*T* *r*_*SU*_	0.04	0.02	0.01	0.02	0.07	0.04	0.03	0.04	0.06	0.05	0.05	0.03	0.05	0.02	0.08	0.07	0.01	0.02	0.06	0.04	0.03	0.02	0.08	0.05
*T* *r*_*US*_	0.02	0.03	0.02	0.02	0.05	0.03	0.04	0.04	0.04	0.04	0.08	0.08	0.04	0.03	0.06	0.06	0.02	0.04	0.09	0.08	0.02	0.03	0.08	0.04
*T* *r*_*SUS*_	0.03	0.02	0.01	0.01	0.05	0.03	0.02	0.02	0.03	0.02	0.02	0.01	0.05	0.03	0.05	0.04	0.01	0.01	0.05	0.03	0.03	0.02	0.01	0.04
*T* *r*_*USU*_	0.01	0.02	0.03	0.03	0.03	0.03	0.04	0.02	0.02	0.03	0.03	0.03	0.03	0.02	0.03	0.03	0.01	0.02	0.08	0.07	0.02	0.02	0.08	0.04
Detection rate																								
*T* *r*_*SU*_	10 %		95 %		100 %		100%		40 %		0 %		100 %		25 %		85 %		100 %		100 %		100 %	
*T* *r*_*US*_	70 %		85 %		95 %		100%		65 %		25 %		45 %		60 %		85 %		100 %		65 %		100 %	
*T* *r*_*SUS*_																								
S →U	10 %		95 %		95 %		95%		30 %		0 %		95 %		35 %		75 %		100 %		95 %		100 %	
U →S	0 %		0 %		95 %		75%		0 %		0 %		0 %		0 %		0 %		0 %		35 %		100 %	
*T* *r*_*USU*_																								
S →U	60 %		70 %		100 %		100%		55 %		25 %		35 %		60 %		85 %		95 %		55 %		95 %	
U →S	0 %		0 %		100 %		100%		0 %		0 %		60 %		0 %		5 %		40 %		65 %		100 %	
Average Run Length [days]																								
	mean	sd	mean	sd	mean	sd	mean	sd	mean	sd	mean	sd	mean	sd	mean	sd	mean	sd	mean	sd	mean	sd	mean	sd
*T* *r*_*SU*_	2.50	2.12	17.47	4.56	10.30	3.40	16.25	2.99	6.38	4.34	/	/	13.75	4.00	7.20	4.60	2.94	2.36	10.20	3.53	15.85	3.05	10.10	3.39
*T* *r*_*US*_	5.29	5.38	16.41	3.39	14.89	3.96	14.00	4.53	14.08	5.89	7.80	7.29	16.89	4.78	12.00	5.41	11.18	4.39	14.05	6.55	23.38	2.79	14.85	6.10
*T* *r*_*SUS*_																								
S →U	4.00	1.41	18.95	2.68	13.00	5.42	16.21	5.14	4.33	3.14	/	/	13.58	4.60	6.29	1.98	2.93	2.25	9.20	4.27	15.79	3.60	10.80	4.27
U →S	/	/	/	/	15.63	3.68	11.20	4.38	/	/	/	/	/	/	/	/	/	/	/	/	24.14	2.14	13.15	5.90
*T* *r*_*USU*_																								
U →S	6.50	6.79	14.64	6.06	16.85	6.06	12.60	5.45	12.27	4.58	10.40	9.34	12.57	4.61	8.58	5.62	11.29	6.57	21.37	5.91	21.55	4.11	14.05	5.35
S →U	/	/	/	/	10.95	3.66	15.65	4.22	/	/	/	/	15.08	3.48	/	/	6.00	0.00	13.88	6.75	20.77	3.14	13.25	4.84

Notes

^a^Training simulation scenarios as described in Table 3

^b^Control chart with rational subgroups implemented

^c^Control chart with a reinitialization after three consecutive alarm days

^d^Control chart with rational subgroups and a reinitialization after three consecutive alarm days

Firstly, there is a lower detection rate when using the standard SPC techniques with transitions from stable to unstable as opposed to transitions from unstable to stable. This is mainly due to the small zone which is defined between the control limits based on the stable gait model, combined with the wide variability present in an unstable gait model. It is therefore less likely to have two consecutive days, during the transition period, on which the data points reside at the same side of the upper and lower control limits.

Next, it can be observed that the ARL of the different methods increases when the rational subgroups method is implemented. When implementing this method, days on which a small number of measurements are present have a smaller influence on the calculated CUSUM or EWMA values. Following this, if at the beginning of the transition period some days have fewer measurements they will delay the detection of the transition.

Moreover, the same increase of ARL can be seen when implementing the reinitialization after three consecutive alarm days. This is caused by the reinitialization when three consecutive days trigger a false alarm. The wider control limits that result from this reinitialization increase the number of days needed to detect an actual transition. If, however, no reinitialization is done, subsequent trends could not be detected.

The Standardized CUSUM has the lowest detection rate, making it not suitable for our application. EWMA and Tabular CUSUM both have better detection rates. The detection rates for both EWMA and Tabular CUSUM were further improved by implementing the reinitialization after three alarm days and by using the rational subgroups method. After this, EWMA has slightly better detection rates than Tabular CUSUM. FPR and ARL of both Tabular CUSUM with a reinitialization after three alarm days (TCRA) and EWMA with reinitialization and rational subgroups (ERSRA) are however similar to each other. Both methods were therefore further optimized for our application.

### Optimization

**Fig. 3:**
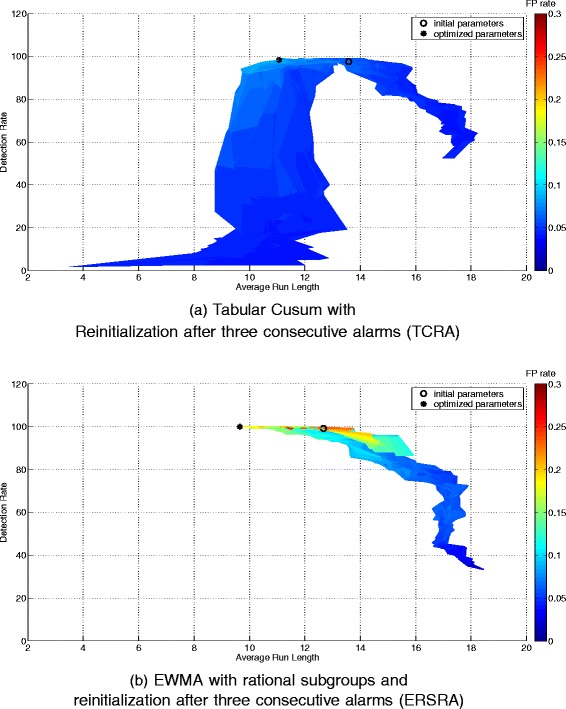
Plot of the average Detection Rate (DR), Average Run Length (ARL) and average number of false alarms (FPR) calculated for a wide variety of parameters over the different simulation scenarios used for parameter optimization. Colors represent the average number of false alarms. The initial parameters were chosen by a rule of thumb (TCRA: *k*=0.5 and *h*=3 and ERSRA: *λ*=0.15 and *L*=3), optimized parameters are chosen based on this plot giving priority to ARL and DR over FPR (TCRA: *k*=0.42 and *h*=2.08 and ERSRA: *λ*=0.18 and *L*=2)

The optimization graphs for both top-ranked methods (Fig. 3) show that a small improvement in detection rates can be reached and that a shorter ARL causes an increase in the average number of false alarms per week. However, since the number of false alarms per week remains very low and due to the fact that a transition from one gait model to another needs to be detected as fast as possible, an improvement in the ARL is desirable. The choice was therefore made to determine a new operating point on these graphs by aiming to raise the detection rate further and to shorten the ARL.

The new operating point, as well as the point resulting from the parameters, as chosen by the rule of thumb, are both plotted on the graphs. The improvements made by changing the operating point are given in Table 6.

**Table 6:** Average results of the best performing methods prior to and after optimization

	TCRA^a^		ERSRA^b^	
	Initial parameters	Optimized parameters	Initial parameters	Optimized parameters
	k = 0.5	k = 0.42	*λ*= 0.15	*λ*= 0.18
	h = 3	h = 2.08	L = 3	L = 2
ARL^c^	13.58	11.07	12.67	9.65
DR^d^	97.50	98.33	99.17	100
FPR^e^	0.06	0.08	0.10	0.18

Notes

^a^Tabular CUSUM with a reinitialization

after three consecutive alarm days

^b^EWMA with rational subgroups and a reinitialization

after three consecutive alarm days

^c^Average Run Length

^d^Detection Rate

^e^Average number of false alarms per week

As both the values for *λ* and *k* remain close to those chosen by the rule of thumb (see Table 6), the improvement in both ARL and DR are mainly due to the lowering of *L* for EWMA and *h* for Tabular CUSUM. This corresponds with lowering the control limits, therefore creating a smaller zone in which variations in the transfer times are considered normal.

Both Table 6 and Fig. 3 show that the highest DR and shortest ARL can be achieved with the EWMA method with rational subgroups implemented and a reinitialization after three consecutive alarm days. *λ* and *L* were optimized into resp. 0.18 and 2. This method will be further validated in the paper.

### Validation

#### Validation on the training set

After optimization the results from the training set were validated using a set of similar simulation scenarios (see Table 4). The results from this validation, shown in Table 7, confirm the prior results for DR as well as ARL and FPR.

**Table 7:** Comparison of the results on training- and validation set (when the dataset contains two trends both trends are evaluated separately)

Training scenarios^a^		Validation scenarios^b^	
Detection Rate (DR)			
*T* *r*_*SU*_	100 *%*	*V*_*SU*_	100 *%*
*T* *r*_*US*_	100 *%*	*V*_*US*_	100 *%*
*T* *r*_*SUS*_		*V*_*SUS*_	
S →U	100 *%*	S →U	100 *%*
U →S	100 *%*	U →S	100 *%*
*T* *r*_*USU*_		*V*_*USU*_	
U →S	100 *%*	S →U	100 *%*
S →U	100 *%*	U →S	100 *%*
Average Run Length (ARL)			
*T* *r*_*SU*_	8.05±3.62	*V*_*SU*_	7.70±3.34
*T* *r*_*US*_	12.95±4.31	*V*_*US*_	10.95±3.59
*T* *r*_*SUS*_		*V*_*SUS*_	
*S*→*U*	7.65±4.10	S →U	8.35±3.59
U →S	11±4.30	U →S	12.45±5.25
*T* *r*_*USU*_		*V*_*USU*_	
U →S	10.15±5.96	U →S	10±3.54
S →U	8±5.58	S →U	8.10±2.88
Average number of false alarms per week (FPR)			
*T* *r*_*S*_	0.15±0.17	*V*_*S*_	0.16±0.07
*T* *r*_*U*_	0.11±0.07	*V*_*U*_	0.14±0.06
*T* *r*_*SU*_	0.20±0.10	*V*_*SU*_	0.20±0.07
*T* *r*_*US*_	0.18±0.07	*V*_*US*_	0.17±0.07
*T* *r*_*SUS*_	0.23±0.08	*V*_*SUS*_	0.20±0.08
*T* *r*_*USU*_	0.23±0.07	*V*_*USU*_	0.20±0.08

Notes

^a^Training simulation scenarios as described in Table 3

^b^Validation simulation scenarios as described in Table 4

#### Variation in transition length

The scenarios from the training set had a transition length of four weeks. The different simulation scenarios were also generated with varying transition lengths of 0, 4, 8 and 12 weeks.

Table 8 demonstrates that a longer transition length causes a longer ARL. Since the model parameters during the transition period are determined through interpolation, a longer transition period causes smaller daily changes and hence it takes longer for the algorithm to detect a significant change. In contrast, when a sudden change in transfer time is present, the ARL is substantially shorter. DR and FPR are not influenced by a change in transition length.

**Table 8:** Validation results of scenarios with varying lengths of transition period

Scenario^a^	Transition period (in weeks)	FPR^b^	DR^c^	ARL^d^
*V*_*SU*_	0	0.16±0.09	95 %	1.74±2.13
	4	0.20±0.07	100 %	7.70±3.34
	8	0.12±0.06	100 %	11.45±5.96
	12	0.16±0.07	100 %	16.30±7.49
*V*_*US*_	0	0.15±0.07	100 %	2.45±1.32
	4	0.17±0.07	100 %	10.95±3.59
	8	0.11±0.06	100 %	15.85±6.75
	12	0.11±0.04	100 %	20.45±11.50
*V*_*SUS*_	0	0.20±0.09	100 %	1.47±0.93
	4	0.20±0.08	100 %	10.40±4.90
	8	0.16±0.05	100 %	15.27±9.30
	12	0.14±0.06	100 %	17.68±10.14
*V*_*USU*_	0	0.18±0.07	100 %	2.25±1.84
	4	0.20±0.08	100 %	9.05±3.33
	8	0.17±0.05	100 %	14.18±7.54
	12	0.15±0.06	100 %	17.25±11.96

Notes

^a^Simulation scenarios as described in Table 4

When two trends are present in the data

an average DR and ARL is calculated

^b^Average number of false alerts per week

^c^Detection Rate

^d^Average Run Length

#### Change in the gait model parameters

Based on the real-life data a change in gait model was characterized by a change in both parameters *μ* and *σ*. A dataset containing transitions characterized by a change in either *μ* or *σ* was also included in the validation set (gait models TGM1 and TGM2).

The results from these datasets compared to those of the training set (see Table 9) demonstrate that a transition characterized by a single change in *μ* yields similar results to those where a change in both parameters was present. If in contrast only *σ* changes during the transition, the detection rate is much lower.

**Table 9:** Comparison of the results on transitions to gait models with varying parameters

Changing parameters	Scenario^a^	FPR^b^	DR^c^	ARL^d^
*μ* and *σ*	*V*_*SU*_	0.20±0.07	100 %	7.70±3.34
	*V*_*US*_	0.17±0.07	100 %	10.95±3.59
	*V*_*SUS*_	0.20±0.08	100 %	10.40±4.50
	*V*_*USU*_	0.20±0.08	100 %	9.05±3.33
*μ* only	*V*_*ST*2_	0.20±0.09	100 %	7.85±2.94
	*V*_*UT*1_	0.16±0.06	100 %	9.55±3.78
	*V*_*ST*2*S*_	0.23±0.04	100 %	8.05±3.41
	*V*_*UT*1*U*_	0.21±0.07	97.50 %	9.97±3.59
*σ* only	*V*_*ST*1_	0.13±0.08	50 %	14.50±6.96
	*V*_*UT*2_	0.09±0.07	25 %	7.20±9.47
	*V*_*ST*1*S*_	0.15±0.06	35 %	11.57±9.07
	*V*_*UT*2*U*_	0.11±0.09	37.50 %	12.87±8.25

This change in gait model parameters is defined as either a change in both *μ*, *σ* or either *μ* or *σ* (all transitions have a length of four weeks)

Notes

^a^Simulation scenarios as described in Table 4

When two trends are present in the data

an average DR and ARL is calculated

^b^Average number of False Alerts per week

^c^Detection Rate

^d^Average Run Length

#### Change in the number of measurements per day

The training set was generated with a varying number of measurements each day (between 0 and 10). A dataset was added to the validation set in which only half the number of measurements was used to generate the data.

Table 10 indicates that a reduction in the number of measurements per day does not influence the performance of the control chart.

**Table 10:** Comparison of the results with a change in the number of measurements per day

Scenario^a^	Number of measurements per day	FPR^b^	DR^c^	ARL^d^
*V*_*SU*_	[0–10]	0.20±0.07	100 %	7.70±3.34
	[0–5]	0.16±0.06	100 %	7.80±4.41
*V*_*US*_	[0–10]	0.17±0.07	100 %	10.95±3.59
	[0–5]	0.16±0.06	100 %	11.05±4.20
*V*_*SUS*_	[0–10]	0.20±0.08	100 %	10.40±4.90
	[0–5]	0.24±0.08	100 %	8.90±4.15
*V*_*USU*_	[0–10]	0.20±0.08	100 %	9.05±3.33
	[0–5]	0.21±0.05	100 %	8.18±4.30

Notes

^a^Simulation scenarios as described in Table 4

^b^Average number of False Alerts per week

^c^Detection Rate

^d^Average Run Length

#### Change in the length of the initialization period

**Fig. 4:**
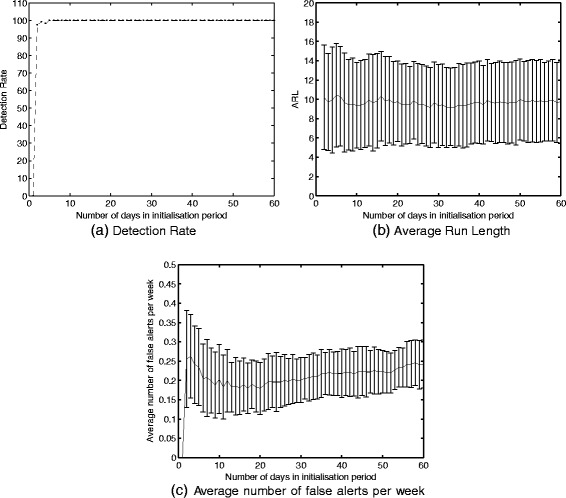
Detection Rate, Average Run Length and Average number of false alerts per week calculated with varying number of days in the initialization period

To assess the necessary length of the initialization period, the ARL, DR and FPR were calculated for all scenarios with a transition period of four weeks and with an initialization period varying from 1 to 60 days. The three criteria were averaged for each initialization period length. This resulted in one value per criteria and per initialization period length. These are shown per criteria in Fig. 4. An optimal Detection Rate is reached with an initialization period of 4 days. For both ARL and the average number of FPR, a longer initialization period is needed. Both, however, show that an initialization period of more than 14 days does not result in an important improvement of the results.

#### Validation on the real-life datasets

After training and validation on simulation data, the algorithm was further validated on the real-life dataset. Three different scenarios were collected during the real-life acquisition. Firstly, two participants were monitored who had a stable gait during the whole acquisition period. Secondly, transfer times of a participant with a very unstable gait during the whole acquisition period were collected. Lastly, a person who transitioned from an unhealthy gait to a healthy gait and transitioned back to an unhealthy gait was monitored.

**Fig. 5:**
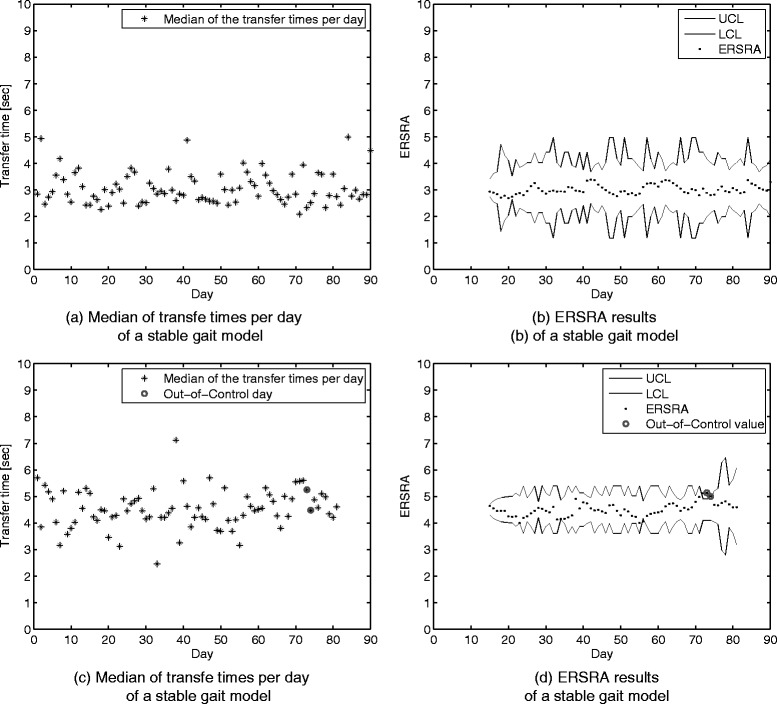
Median of transfer times compared to the EWMA rational subgroups with reinitialization after three consecutive alarm days (ERSRA) results. The first 14 days were used as an initialization period and are therefore not used on the ERSRA control chart

The first participant (Fig. 5(a) and (b)) had a stable gait model during the whole acquisition period and triggered no false alarms during this period.

Although the second participant was reported being in good health during the acquisition period two consecutive alarms were triggered (Fig. 5(c) and (d)). These alarms were caused by the three consecutive days prior to these alarms. On these days, longer transfer times were recorded which could indicate that some health-related problems were present during these days prior to the alarm.

**Fig. 6:**
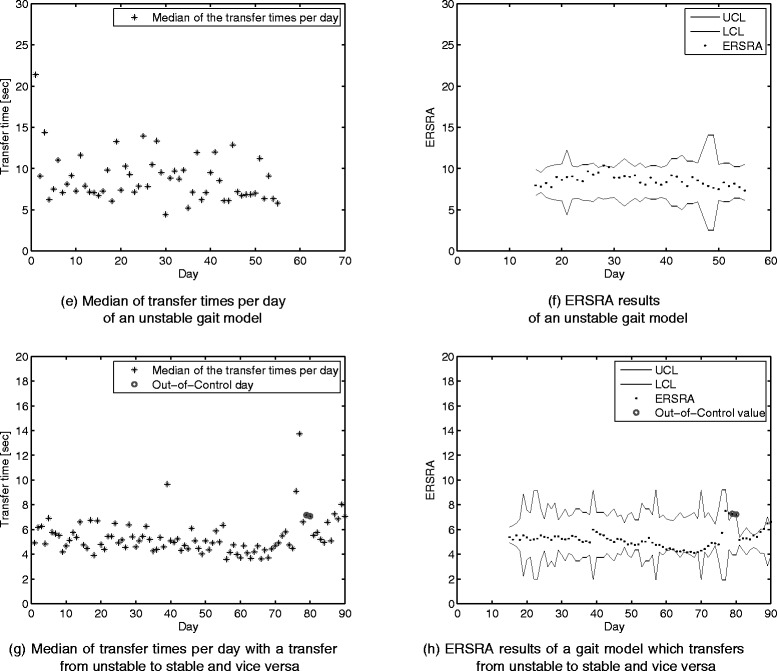
Median of transfer times compared to the EWMA rational subgroups with reinitialization after three consecutive alarm days (ERSRA) results. The first 14 days were used as an initialization period and are therefore not used on the ERSRA control chart

The third participant had a very unstable gait during the whole acquisition period (Fig. 6(e) and (f)). Although this was a very unstable gait, no changes in health were reported and no alerts were triggered by our algorithm.

The last participant started the acquisition period with an unstable gait. The gait improved slightly during the acquisition period, but given that this improvement was quite small, no alerts were triggered. After this improvement, the participant experienced a rapid decline in health after a stroke, which is visible in the measured transfer times. This resulted in two alarms (Fig. 6(g) and (h)).

## Discussion

This study reports on the selection, optimization and extensive validation of a system to automatically detect changes in the health of older adults using transfer times. The best performing method was the EWMA control chart implemented with rational subgroups and a reinitialization after three consecutive alarm days. After optimization, the selected method had a detection rate of 100 % and an average run length of 9.65 days when the length of the transition period was 28 days. These results were confirmed using a separate validation dataset, confirming the suitability of the presented method for the application at hand.

There are still some aspects of the system that warrant further clarification.

### The design process

The tabular CUSUM, standardized CUSUM and EWMA control charts were evaluated because of their ability to detect small shifts in the data and their good performance with skewed distributed data. These control charts, however, still assume that the data is normally distributed. Some control charts can be optimized for a log-logistic distribution such as the Shewart Chart and the Range Chart. These charts are in comparison with the EWMA and CUSUM control charts, less suited to detect small shifts. They were therefore not incorporated in this research.

To evaluate the different control charts simulated data, generated based on previously acquired real-life data was used. The number of real-life datasets was limited. When generating simulated data some scenarios which did not fit the real-life data were also incorporated to reduce the risk of over-fitting on the real-life data. Theoretical gait models were incorporated in these scenarios as well as different transition period lengths and a varying number of measurements per day.

Furthermore, in previous studies it was shown that some noise is present in the measured transfer times. This noise is due to imperfect preprocessing steps and changes in the used walking aid [21]. To reduce the influence of noisy measurements on the control charts a median was calculated per day and used as input for the control charts. The rational subgroups methods was also implemented to reduce the influence of a day with a small number of measurements on the performance of the control charts and therefore reducing the number of false alarms.

The Western Electric rules were implemented to further improve the performance of the control charts. These are decision rules used for the detection of out-of-control conditions on control charts. Since the desired goal was the detection of process instabilities the so-called zone rules were applied. These rules specify that a process is out of control when two or three consecutive points fall beyond a predefined zone [30]. Based on this an alert is triggered when either the positive/negative CUSUM value or the EWMA value is outside the Control Limits for at least two consecutive days.

Lastly, a reinitialization after three consecutive alarm days was implemented to facilitate the detection of subsequent trends.

### Strengths and weaknesses of the presented method

Based on the simulated data, the proposed system will report one false alert every five weeks. Although some of these false alarms are due to outliers in the generated data and hence still of interest, the system should under ideal conditions produce less false alerts whilst maintaining the current detection rate. When one would widen the control limits to decrease the number of false alerts, the ARL will generally lengthen and the detection rate will decrease. Similarly, when the control limits are tightened, the opposite happens. However, since a false alert is triggered when the measurements of two subsequent days are substantially different to those of the previous days, it could indicate that some health problems are apparent during those days. Although no transition is present when such an alarm is triggered, a visit of a healthcare professional might still be needed. A compromise was therefore sought between detection rate, ARL and the average number of false alerts per week.

When using the presented methodology, it is assumed that all values between the Upper Control Limit and the Lower Control Limit are good. However, a value closer to the Central Line is better than one closer to the Control Limits. It could therefore be useful to trigger a first alarm if on several subsequent days the time points reside close to the Control Limits. This can be done by implementing more of the Western Electric zoning rules. These rules also describe when an alarm should be triggered, even when a time point lies between the Control Limits, depending on its distance to the Central Line and the previous points.

A transfer could remain undetected when a transition period starts and ends during the initialization period. If it starts at the end of the initialization period and ends after the initialization period, the transition, however, can still be detected depending on the length of the transition period and the change in *μ* after the initialization period. The number of days needed to detect the transition will increase. It is therefore advised to keep the initialization period as short as possible.

The major strength of the presented method is that it is a generic method. Although the presented research monitored changes in transfer times, it could equally well be applied to gait speed or other quality characteristics such as step length, stride length and activity level. However, a new optimization phase might be necessary to find the optimal values for both *λ* and *L*.

## Conclusion

The system presented in this paper is able to detect both positive and negative trends in the transfer times of older adults, therefore automatically triggering an alarm when gradual and abrupt changes in transfer times, which are closely linked to gait speed, occur. Since previous research has shown that changes in gait speed can have a predictive value for a broad array of adverse events, several research groups have already designed and validated systems which can measure the gait speed of a person on a daily basis. They however do not trigger alerts and a healthcare worker is needed to review the data. It is therefore anticipated that the presented technique can provide a valuable addition to existing gait monitoring systems.

## Availability of supporting data

The simulated data generator was built using MathWorks’ Matlab. The code will become available for download on the website: www.kuleuven.be/advise/datasets. Due to ethical regulations the real-life datasets are not publicly available.

## Abbreviations

ARL: average run length
CUSUM: CUmulative SUM control chart
CL: central line
DR: detection rate
EWMA: exponentially weighted moving average (EWMA) control chart
E: EWMA
ERA: EWMA reinitialize alarm
ERS: EWMA rational subgroups
ERSRA: EWMA reinitialize alarm rational subgroups
FPR: false positive rate
LCL: lower control limit
SPC: statistical process control
SC: standardized CUSUM
SCRA: standardized CUSUM reinitialize alarm
SCRS: standardized CUSUM rational subgroups
SCRSRA: standardized CUSUM reinitialize alarm rational subgroups
SGM: stable gait model
TC: tabular CUSUM
TCRA: tabular CUSUM reinitialize alarm
TCRS: tabular CUSUM rational subgroups
TCRSRA: tabular CUSUM reinitialize alarm rational subgroups
TGM: theoretical gait model
TGUG: timed-get-up-and-go
UCL: upper control limit
UGM: unstable gait model
